# Isolation and Characterization of the Novel Phages vB_VpS_BA3 and vB_VpS_CA8 for Lysing *Vibrio parahaemolyticus*

**DOI:** 10.3389/fmicb.2020.00259

**Published:** 2020-02-21

**Authors:** Meiyan Yang, Yongjian Liang, Shixuan Huang, Jumei Zhang, Jing Wang, Hanfang Chen, Yuanming Ye, Xiangyang Gao, Qingping Wu, Zhiyuan Tan

**Affiliations:** ^1^College of Agriculture, South China Agricultural University, Guangzhou, China; ^2^State Key Laboratory of Applied Microbiology Southern China, Guangdong Provincial Key Laboratory of Microbiology Culture Collection and Application, Guangdong Open Laboratory of Applied Microbiology, Guangdong Institute of Microbiology, Guangzhou, China; ^3^Guangdong Provincial Key Laboratory of Nutraceuticals and Functional Foods, College of Food Science, South China Agricultural University, Guangzhou, China

**Keywords:** *Vibrio parahaemolyticus*, *Vibrio* phage, *Siphoviridae*, lytic bacteriophages, biocontrol, aquaculture

## Abstract

Accumulating evidence has indicated that the multiple drug resistant *Vibrio parahaemolyticus* may pose a serious threat to public health and economic concerns for humans globally. Here, two lytic bacteriophages, namely vB_VpS_BA3 and vB_VpS_CA8, were isolated from sewage collected in Guangzhou, China. Electron microscopy studies revealed both virions taxonomically belonged to the *Siphoviridae* family with icosahedral head and a long non-contractile tail. The double-stranded DNA genome of phage BA3 was composed of 58648 bp with a GC content of 46.30% while phage CA8 was 58480 bp with an average GC content of 46.42%. In total, 85 putative open reading frames (ORFs) were predicted in the phage BA3 genome while 84 were predicted in that of CA8. The ORFs were associated with phage structure, packing, host lysis, DNA metabolism, and additional functions. Furthermore, average nucleotide identity analysis, comparative genomic features and phylogenetic analysis revealed that BA3 and CA8 represented different isolates but novel members of the family, *Siphoviridae*. Regarding the host range of the 61 *V. parahaemolyticus* isolates, BA3 and CA8 had an infectivity of 8.2 and 36.1%, respectively. Furthermore, ∼100 plaque-forming units (pfu)/cell for phage BA3 and ∼180 pfu/cell for phage CA8 were determined to be the viral load under laboratory growth conditions. Accordingly, the phage-killing assay *in vitro* revealed that phage CA8 achieved approximately 3.65 log unit reductions. The present results indicate that CA8 is potentially applicable for biological control of multidrug resistant *V. parahaemolyticus*.

## Introduction

The Food and Agriculture Organization of the United Nations (FAO) reported that gains sufficient and nutritious food is jeopardized by the rapid increase in the human population (FAO, 2019a). As a result, aquaculture, the farming of aquatic organisms, has been a crucial activity that has contributed to aquatic-sourced foods for national food security and economic development. Currently, valuable aquatic species, such as farmed shrimp, marine fish, and freshwater fish, are considered as commodities for the export market and monetary gain. Total production of global farmed shrimp reached almost 4 million ton in 2018, increasing by 3 to 5% over 2017 (FAO, 2019a). Global fish production was also estimated to increase by 2.1% to 178.8 million ton in 2018, with 87 million tons (48.7%) being derived from aquaculture and 91.8 million tons (51.3%) from capture fisheries (FAO, 2019a). Such substantial change in the aquaculture sector resulted in the amount of wild capture fisheries for human consumption being surpassed. Moreover, consumer behaviors have become increasingly universal, especially in developed countries and China. For example, the US per capita shrimp consumption is bound to be historically high, crossing the 4.5-lb maximum in 2018 (FAO, 2019a). Mainland China produced 66.8 million ton of fish for human consumption in 2016, with 49.2 million ton (74%) from aquaculture and 17.6 million ton (26%) from wild capture (FAO, 2019b).

Unfortunately, aquaculture-associated bacterial diseases, which are typically caused by *Vibrio parahaemolyticus*, are considered to be a challenge to food safety, economic development, and human health. *V. parahaemolyticus* is a gram-negative bacterium that has been commonly associated with infections in aquatic organisms in aquaculture farm and the wild. The cost of such disease is significantly greater than previous official estimates and demonstrates the serious problems exerted by foodborne illnesses (commonly known as food poisoning) on social and economic systems. Anyone who consumes raw or undercooked fish, shrimp, shellfish, and other seafood is at risk of contracting *V. parahaemolyticus* (Thompson et al., 2004). Foodborne illness, which is closely related to *V. parahaemolyticus*, is often caused by consuming food contaminated by bacteria and their toxins, such as thermostable direct hemolysin (TDH), TDH-related hemolysin (TRH), and type III secretion system 1 (T3SS1) and system 2 (T3SS2) (Raimondi et al., 2000; Naim et al., 2001; Park et al., 2004; Hiyoshi et al., 2010; Letchumanan et al., 2014; Paria et al., 2019). Most people infected with *V. parahaemolyticus* develop symptoms such as diarrhea, vomiting, abdominal cramps, nausea, fever, and stomach pain after approximately 24 h. Moreover, diarrhea tends to be watery and is occasionally bloody. In total, 26 laboratory-confirmed cases of *V. parahaemolyticus* infections were reported in people who ate fresh crab meat from Venezuela on September 27, 2018 (FDA, 2018). When penicillin was discovered in the 1920s, antimicrobial agents were a crucial practice in animal therapy (Aarestrup and Wegener, 1999). Currently, to rapidly and effectively control and terminate the proliferation of pathogenic *Vibrio* sp., the prophylactic and therapeutic use of antibiotics has been widely employed in hatcheries, the transportation and sales chain; this is despite urgent concerns regarding the development and dispersal of multiple antibiotic-resistance in the pathogen community. Indeed, microbiological risk assessments revealed that both aquatic products and ready-to-eat foods in China can be contaminated by antimicrobial-resistant *V. parahaemolyticus*, with the highest resistance rates observed against streptomycin (Xie et al., 2015, 2016, 2017; Xu et al., 2016). Evidently, as antibiotic-resistant strains cause treatment failure, inducing the violent death of these bacteria is a challenge. Increasing researches have indicated that the multiple drug resistant *V. parahaemolyticus* may pose an especially serious threat to public health and economic concerns for humans globally (Lesmana et al., 2001; Ottaviani et al., 2013; Kang et al., 2017). To prevent and control contaminated *V. parahaemolyticus* in aquatic products, an effective practice instead of the abuse of antibiotics is an evident resolution for the economy and food safety.

Bacteriophages (phages) are the most abundant type of biological particles on Earth. They are composed of either DNA or RNA enclosed in a protein coat (Hendrix, 2003). Phage genomes contain a very high proportion of novel genetic sequences of unknown function, and may represent the largest reservoir of unexplored genes (Hatfull, 2008). Unlike antibiotics, lytic phage multiplication in the host (attachment, penetration, biosynthesis, maturation, assembly, and release) can control and kill the bacterium (Yang Z. et al., 2019). With the inevitable and frequent occurrence of drug-resistant pathogenic bacteria and the sharp decline in novel antibiotic discovery, research, and application, phage therapy is regaining attention after years of continuous worsening of antibiotic resistance owing to the great potential of cell disruption by bacteria (Carlton, 1999; Lu and Collins, 2007, 2009). Nowadays, several studies for identification and characterization as well as animal application of *Vibrio* phages have been carried out, including phages pVp 1, A3S, Vpms1, VP-1, VP-2, VP-3, VPp1, Vpp1, Vpp2, Vpp3, Vpp4, Vpp5, Vpp6, VP1, VP7, VP9, OWB, vB_VpaP_VP-ABTNL-1, and vB_VpaS_VP-ABTNL-2 (Jun et al., 2014; Lomelí-Ortega and Martínez-Díaz, 2014; Mateus et al., 2014; Rong et al., 2014; Alagappan et al., 2016; Chang et al., 2016; Zhang et al., 2016; Ren et al., 2019). Therefore, phage therapy is considered to be an environmentally friendly practice with huge potential to reduce and control drug-resistant *V. parahaemolyticus* in aquaculture.

## Materials and Methods

### Bacterial Strains and Phage Isolation

All *V. parahaemolyticus* strains in our work were provided by the Guangdong Institute of Microbiology and stored at −80°C in 15% (v/v) glycerol and cultivated at 37°C, with shaking at 200 rpm in Trypto soya broth (TSB). The *Vibrio* phages were isolated from sewage samples collected in the Huangsha aquatic product market, from the host, *V. parahaemolyticus* O1-1 and O3-11. Phage concentration from the fluid sample was used for membrane filter adsorption and elution as described before (Van Twest and Kropinski, 2009). The water sample was centrifuged at 8000 × *g* for 10 min at room temperature and filtered (0.45-μm syringe filter) to remove large particulates and bacteria. Solid MgSO_4_ was added to a final concentration of 50 mM and the mixture was filtered slowly through the 0.45-μm (47 mm diameter) mixed cellulose ester GSWP filter. Before placement in the ultrasonic cleaning bath for 10 min, the filter was cut into many pieces, which were then placed in a flask with 50 mL of eluting solution [1% (w/v) Bacto beef extract and 3% (v/v) Tween 80]. To culture the lytic phage of the target host, the filtered phage concentrate (2.5 mL) was mixed with 2.5 mL of sterile double-strength TSB (4 mM CaCl_2_) and 100 μL culture (OD_600_ ∼0.2) of *V. parahaemolyticus* isolate (O1-1/O3-11), and then incubated at 37°C for 24 h with shaking. The mixed culture was then centrifuged at 8000 × *g* for 5 min at room temperature and the supernatant was filtered (0.45-μm syringe filter) to remove residual bacterial cells. The process of phage enrichment was repeated at least three times. A 100-μL diluted sample of the phage culture was mixed with 100 μL of *V. parahaemolyticus* cells (mid-log phase) and incubated at 37°C for 15 min. Approximately 5 mL of the top agar (TSB with 0.4% agar) at 45°C was then added, and the mixture was poured onto a TSA plate. The plates of phage BA3/CA8 were then incubated at 37°C for 5 h/8 h to obtain phage plaques. Purification of the two phages was performed at least 10 times. Lastly, purified and targeted phages were stored at 4°C in TSB for 1 month and −80°C in 30% (v/v) glycerol.

### CsCl Gradient Purification and Electron Microscopic Analysis

Isopycnic centrifugation through CsCl gradients was performed as previously described, with modifications (Ajuebor et al., 2018). A high titer phage lysate was precipitated using polyethylene glycol (15% w/v PEG8000, 0.5M NaCl) at 4°C overnight and centrifuged at 12000 × *g* for 20 min at 4°C. Thereafter, the pellet was resuspended in SM buffer. The resulting phage preparation was placed on a CsCl step gradient composed of 1.3, 1.5, and 1.7 g/mL layers and spun in a SW 41 Ti rotor (Optima XPN-100 ultracentrifuge, Beckman Coulter, Brea, CA, United States) at 200600 × *g* (34200 rpm) for 3 h at 4°C. The collected phage could be used for electron microscopic analysis. The prepared phages were placed on freshly-prepared carbon films with 2% (w/v) uranyl acetate. Morphological characteristics were observed under a transmission electron microscope (TEM, Hitachi H-7650) at an acceleration voltage of 80 kV with a CDD camera.

### DNA Extraction and Genome Sequencing

Genomic DNA was extracted from BA3/CA8 based on a method described in a previous work, with some modifications (Xing et al., 2017). In brief, DNase I and RNase A were added to the phage preparation to a final concentration of 0.1 Units/μL and 3 μg/mL. The mixture was incubated for 1 h at 37°C. After subsequent treatment with SDS, EDTA, and proteinase K, the mixture was further incubated for 30 min at 65°C. An equal volume of phenol was added for DNA extraction. After centrifugation at 12000 × *g* for 5 min at room temperature, the aqueous layer was transferred to a fresh tube containing an equal volume of phenol-chloroform-isoamyl alcohol (25:24:1) and then centrifuged at 12000 × *g* for 5 min at room temperature. The aqueous layer was collected, mixed with an equal volume of isopropanol, and stored for 30 min at −20°C. The mixture was then centrifuged at 4°C for 20 min at 12,000 × *g*, and the DNA pellet was washed twice with 75% ethanol. Lastly, the DNA was air dried at room temperature, resuspended in deionized water, and stored at −20°C.

To obtain the viral DNA required for high-throughput sequencing (HTS), the absence of free and contaminating bacterial DNA was validated via PCR amplification of the bacterial 16S rRNA gene with universal primers, 27F/1492R (Jin et al., 2019). Thereafter, the extracted BA3/CA8 genomic DNA was sequenced using the semiconductor sequencer in an Ion Torrent^TM^ Personal Genome Machine. This technology used emulsion PCR and a sequencing-by-synthesis approach. The integrity and size of DNA preparation were validated by gel electrophoresis. Sonication of genomic DNA was performed on the Covaris ^®^ S2 or S220 Systems to generate DNA fragments suitable for 500-base-read libraries for end-repair. These DNA fragments were then ligated to Ion Xpress^TM^ Barcode Adaptors Kits for subsequent nick repair and purification with Agencourt ^®^ AMPure ^®^ XP Reagent. Purified DNA fragments of ∼500 bp were selected by using E-Gel ^®^ SizeSelect^TM^ agarose gel. After amplification and purification of the selected library, emulsion PCR was used for processing. PCR was performed in a water-in-oil microreactor containing a single DNA molecule on a bead. The H^+^ Ion Torrent^TM^ signal was detected during the sequencing-by-synthesis process. High quality reads were assembled using the SPAdes v. 3.6.2 program (Bankevich et al., 2012).

### Bioinformatic Analysis

Before complete genome analysis, DNA terminus was identified by CLC Genomics Workbench to initially derive high-frequency sequences (HFSs) in next generation sequencing reads. Briefly, HTS data were aligned to the reference assembly using a CLC Genomics Workbench. The CLC module was used with default parameters (mismatch cost 2, insertion cost 3, deletion cost 3, length fraction 0.5, and similarity 0.8) using FASTQ files as the read input and FASTA files as the reference input. Furthermore, High Occurrence Read Termini theory was performed to study the phage genome termini and support the result (Zhang et al., 2018). Firstly, nucleotide sequence data of the phage was analyzed with the nucleotide BLAST search program^1^ at the National Centre for Biotechnology Information (NCBI). Furthermore, the average nucleotide identity (ANI) was determined among all pairwise combinations of phage genomes, using the BLASTN alignment tool in the pyani package and plotted in an interactive heatmap using heatmaply to identify phages (Pritchard et al., 2016). Secondly, a putative open reading frame (ORF) was automatically predicted by Prokka 1.1.3 and functional annotation was re-screened by BLASTp against the Non-Redundant Protein Database of NCBI, where the score was set > 50 and e-value < 1.0 × 10^–3^ (Seemann, 2014). The functional protein characteristics were predicted with the proteomic tools, InterPro (Mitchell et al., 2019). The virulence signature was determined using the Virulence Factor Database (Liu et al., 2019). A coverage of 95% and identity of 90% for any sequence were required. Artemis Comparison Tool (ACT) was used for comparison to other phages in GenBank and identification of feature variations between the genomes of phages, with homology assessed using BLASTN (Carver et al., 2005). Global genome comparison map among available phage data in NCBI were visualized by the Easyfig 2.2.3 visualization tool (Sullivan et al., 2011). Lastly, based on the major capsid protein and terminase large subunit, phylogenetic analysis was performed with MEGA X by the neighbor-joining method with 1000 bootstrap replicates (Kumar et al., 2018).

### Identification of Proteins Associated With Phage Virions

Samples of the phage were added to the RIPA lysis buffer for protein extraction. Fifty micrograms of protein was transferred into Microcon devices YM-10 (Millipore) and centrifuged at 12000 × *g* at 4°C for 10 min. Subsequently, 200 μL of 50 mM ammonium bicarbonate was added to the concentrate, followed by centrifugation; this step was repeated once. After being reduced by 10 mM DTT at 56°C for 1 h and alkylated by 20 mM IAA at room temperature in dark for 1 h, the mixture was centrifuged at 12000 × *g* at 4°C for 10 min and wash once with 50 mM ammonium bicarbonate. Hundred microliters of 50 mM ammonium bicarbonate and free trypsin were added into the protein solution at a ratio of 1:50, and the solution was incubated overnight at 37°C. The mixture was centrifuged at 12000 × *g* at 4°C for 10 min. Hundred microliters of 50 mM ammonium bicarbonate was added into the device and centrifuged; this step was repeated once. The extracted peptides were lyophilized to near dryness and subsequently resuspend in 50 μL of 0.1% formic acid before LC-MS/MS analysis. The raw MS data were analyzed and searched against the target protein database, based on the species of the samples using MaxQuant 1.6.2.10. The experiment was repeated three times for each phage.

### Phage Titer Assay

Determining the concentration of infectious phage particles is a fundamental protocol for researchers working with bacterial viruses. Phage titer for each experiment was determined by the double agar overlay plaque assay as previously-described with some modifications (Kropinski et al., 2009). Briefly, a 100-μL dilution of the phage culture and 100 μL of *V. parahaemolyticus* cells (mid-log phase) were added to 5 mL of top agar (2 mM CaCl_2_) at 45°C. To form more obvious phage plaques, 0.2% of agar was used in this top plate. Thereafter, the mixture was poured onto a TSA plate and incubated at 37°C for 5 h/8 h to obtain phage plaques for phage BA3/CA8. This assay of suitable dilution was performed with three replicate plates for each dilution. Lastly, we determined the titer of the original phage culture by using the following calculation formula: number of plaques × 10 × reciprocal of counted dilution = pfu/mL.

### Phage Host Range Determination

A host range assay was performed for phages BA3 and CA8 using a previously-described protocol for the spot assay with some modifications (Kutter, 2009). Briefly, 100 μL of bacteria in the mid-log phase and 5 mL of TSB containing 0.4% agar (2 mM CaCl_2_) were mixed and poured onto the TSA plate. After a 5-min standing at room temperature, 2 μL of the 10-fold serially diluted phages in TSB were spotted onto TSB soft agar. The plates were then incubated at 37°C and examined for plaques after 3–24 h. Bacterial sensitivity to a phage was established by a lysis cleared zone at the spot. Based on the clarity of the spot, bacteria were divided into two categories: clear lysis zone (+) and no lysis zone (−).

### Optimal Multiplicity of Infection (MOI) Determination and One-Step Growth Assays

To determine the optimal MOI, serial dilutions of *V. parahaemolyticus* O1-1/O3-11 grown to its exponential phase were added to Li et al. (2019) aliquots of a stock solution of BA3/CA8. Based on the proportion of infection, ratios of 0.0001, 0.001, 0.01, 0.1, 1, 10, and 100, plus 100 μL of phage and 100 μL of *V. parahaemolyticus* (1 × 10^8^ cfu/mL) were added to 5 mL of TSB (2 mM CaCl_2_). Thereafter, the mixtures were shaken at 37°C for 10 h, centrifuged at 8000 × *g* for 5 min at room temperature for cell removal. The precipitate was then removed and the supernatant was filtered through a 0.45-μm membrane. The titer of the phage was determined by a soft agar overlay method. The one which obtained the highest phage titer was the optimal MOI. Three parallel experiments were performed for this MOI test.

The one-step growth curve was generated according to a previous report with minor modifications (Yin et al., 2019). *V. parahaemolyticus* culture (5 mL) was incubated to OD_600_ = 0.2 and the bacterial cell suspension was diluted to 1.0 × 10^8^ cfu/mL. Thereafter, the bacterial cells (1 mL) were pelleted via centrifugation (8000 × *g*, 5 min, room temperature), re-suspended in SM buffer (0.9 ml), and mixed with 0.1 mL of a 1.0 × 10^8^ pfu/mL phage suspension (MOI = 0.1, 2 mM CaCl_2_). The mixture was incubated for 15 min at 37°C. After centrifugation (12,000 × *g*, 2 min, room temperature), the supernatant with free phage was removed and the pellet was suspended in 10 mL TSB (2 mM CaCl_2_). The mixture was then incubated at 37°C with shaking (200 rpm). Every 10 min, the phage titer was determined via the double-layer agar technique. Burst size was calculated as the ratio of the final count of liberated phage particles to the initial count of infected bacterial cells during the latent period.

### Resistance to Environmental Stresses

Heat and pH stability tests were conducted for the phages following a previously-described protocol with some modifications (Laemmli, 1970). Phage suspension (1 × 10^8^ pfu/mL) for each temperature was carried out in a 1.5-mL tube and kept at different water bath temperatures (20, 37, 40, 50, 60, 70, and 80°C) for 1 h. Phage suspensions were incubated at the corresponding selected temperatures. To determine phage pH stability, a gradient of pH was derived to range from 3 to 11. For this experiment, phage suspension (1 mL) was added to the tryptic soy broth (9 mL) with a specific pH followed by incubation at 37°C for 1 h. Three parallel experiments were performed for the stability of the two phages and phage titer was determined via the double-layer agar technique.

### *In vitro* Phage-Killing Assay

Bacterial killing assays were conducted for the phages following a previously-described method with some modifications (Yen et al., 2017). Briefly, 1 mL overnight cultures of *V. parahaemolyticus* isolate (O1-1/O3-11) were added into 50 mL TSB (2 mM CaCl_2_) and grown to 10^8^ cfu/mL at 37°C with shaking (200 rpm), respectively. Bacterial cultures were mixed with phage lysates (MOI 0.1) and incubated for 15 min at 37°C. After that, The mixture was then incubated at 37°C with shaking (200 rpm). At each time point, bacteria were collected, serially diluted, plated onto TSA, and incubated at 37°C for 24 h. Colonies were enumerated to calculate *V. parahaemolyticus* cfu/mL. Each condition was assessed in triplicate.

### Statistical Analysis

Data are expressed as means and standard deviation values. GraphPad Prism 8.0.1 was used for statistical analysis.

#### Nucleotide Sequence Accession Number

The sequence data for the *Vibrio* phages, vB_VpS_BA3 and vB_VpS_CA8, were deposited at GenBank under accession numbers, MN175679 and MN102376.

## Results and Discussion

### Origin and Morphology of Phages BA3 and CA8

Several water samples from aquatic product markets were collected and tested for the presence of bacteriophages against *V. parahaemolyticus* isolates. Based on the clear phage plaque on the plate, the targeted phages were purified using the double-layer agar technique. In this study, phages with lytic activity, designated as vB_VpS_BA3 (phage BA3) and vB_VpS_CA8 (phage CA8), were isolated using the double-layer agar assay technique. Our results suggested that phages can be found in virtually all places where their related hosts exist. Several other investigations reported the presence of *Vibrio*-specific lytic phages in environmental water (shrimp ponds, aquaculture system, sea water and aquatic product market) from different regions of the world (Alagappan et al., 2010; Luo et al., 2016; Stalin and Srinivasan, 2016; Katharios et al., 2017; Srinivasan and Ramasamy, 2017; Yang M. et al., 2019).

**FIGURE 1:**
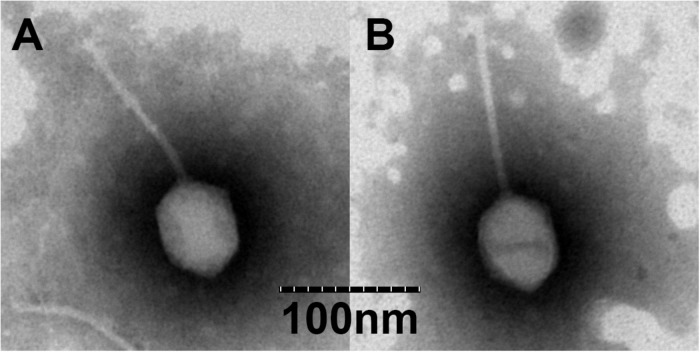
Transmission electron microscopy (TEM) of vB_VpS_BA3 **(A)** and vB_VpS_CA8 **(B)**.

To date, bacteriophages are classified based on differences in the morphology of their virion. Results from TEM revealed that BA3 (Figure 1A) and CA8 (Figure 1B) consist of an icosahedral head and a long non-contractile tail. The measurements of head length, head diameter, and tail length of BA3 were 70, 55, and 130 nm while those of CA8 were 70, 55, and 125 nm. By combining their morphological characteristics with the formal guidelines of the International Committee on Taxonomy of Viruses, phages BA3 and CA8 exhibited typical characteristics of phages belonging to the family, *Siphoviridae*, in the order, *Caudovirales* (Lefkowitz et al., 2018). A similar morphological observation was reported for the *Vibrio* phage, VpKK5 (Lal et al., 2016). In previously-reported scientific literature, over 95% of the identified phages were classified as *Caudovirales* (tailed phages). Meanwhile, almost (60%) of all described that phages with long and flexible tails should be assigned to the family, *Siphoviridae*. Researches have focused on phages infecting various foodborne pathogens majorly including *Listeria monocytogenes*, *Escherichia coli*, and *Salmonella* phages. A study focusing on *Vibrio* phages in *Siphoviridae* showed that it was far from enough compared to *Podoviridae* and *Myoviridae* (Chibani et al., 2019). However, related reports of phage therapy mainly and currently focus on *Caudovirales*. Some reports even highlighted that *Siphoviridae* may adapt a broader host range than *Podoviridae*. Therefore, the isolation and characterization of novel phages, especially *Siphoviridae*, are necessary before biocontrol can be achieved for the multidrug resistant *V. parahaemolyticus*.

### Host Range Test Analysis

To determine the role of *V. parahaemolyticus* in phage infectivity, we performed a spot assay using 61 *V. parahaemolyticus* strains (11 groups of O antigen). Results showed that phage BA3 infected 5 of the 61 isolates (O1, O2, O3, and O5). Thus, we could conclude that the phage-host range was quite narrow. However, 22 strains of *V. parahaemolyticus* (O1, O2, O3, O4, O5, O8, O9, O10, O11, and O12) were sensitive to lysis by phage CA8 (Supplementary Table S1). The lysis effect (22/61) revealed that phage CA8 had the potential to be a candidate for phage therapy. Furthermore, we could predict that phage BA3 and phage CA8 were not cohesive or identical based on the great difference in their host range (Chibani et al., 2019).

### Optimal MOI and One-Step Growth Curve

**FIGURE 2:**
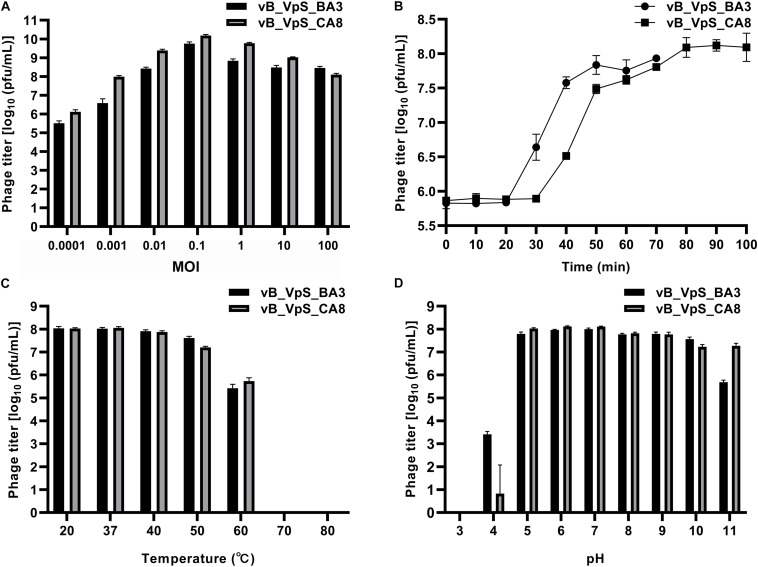
Biological characterization of vB_VpS_BA3 and vB_VpS_CA8. **(A)** Multiplicity of infection; **(B)** Kinetics of progeny production in the single life cycle; **(C)** Thermal stability of two phages treated with different temperature for 1 h; **(D)** pH stability of two phages treated with different pH for 1 h. Results are presented as mean values ± SD and shown as log_10_ (pfu/mL).

As shown in Figure 2A, the *V. parahaemolyticus* strain, O1-1/O3-11, when infected at an MOI of 0.1, generated the maximum bacteriophage titer, indicating that 0.1 was the optimal MOI for the phage BA3/CA8 titer. Owing to the largest titer for the MOI of the two phages, we investigated the latency period and burst size of phages and the results showed in Figure 2B. The assays revealed latent periods, defined as the time interval between the adsorption and the beginning of the first burst, of ∼20 min (phage BA3) and ∼30 min (phage CA8). It was determined to be ∼100 plaque-forming units (pfu)/cell for phage BA3 and ∼180 pfu/cell for phage CA8 under the laboratory growth conditions. Previously, the burst size of the phage VP06 infecting strain, YAF1206-1, was reported to be approximately 60 pfu/cell, with a latent period of ∼30 min (Wong et al., 2019). Another phage, SIO-2, could readily propagate on SWAT3 with a latent period of approximately 45–60 min and average burst size of 60 viral particles per infected cell (Baudoux et al., 2012). The latent period of the *Vibrio* phage, VpKK5, was short at 24 min but exhibited a large burst size of 180 pfu per infected cell (Lal et al., 2016). Compared to the three phages reported, phage BA3 and CA8 exhibited a short latent period and a large burst size (∼100 pfu per infected cell). Latent period and burst size are critical and necessary in the assessment of phage fitness and identification of a candidate for phage therapy. Thus, our result reflects the lytic effectiveness of both phages toward the *V. parahaemolyticus* isolates.

### Resistance to Environmental Stresses

The resistance to environmental stresses assay was performed to investigate the phage application of primary conditions and future prospects (Krasowska et al., 2015). First, phage stability following exposure to varying temperatures and pH was determined. As shown in Figure 2C, both phages BA3 and CA8 were stable at 20 and 37°C. Although, the titer of the phages was slightly reduced after 1 h of exposure at 40°C, these phages were stable at temperatures ranging from 20 to 40°C. Incubation at more than 60°C for 1 h was lethal to the phages, thus completely inactivating them. Such characteristics aligned with the results reported in a previous study with the phage VpKK5. Every phage has an optimal pH for survival and biological activity. As shown in Figure 2D, phage CA8 had optimal stability from pH 5.0 to pH 7.0 while BA3 was stable between pH 6.0 and pH 7.0. When the pH level was decreased, the titer of the two phages was sharply reduced, and at pH 4.0, the phages were essentially inactive. When the pH level increased to 10 and 11, only a low titer of these phages existed and survived. Therefore, we can infer that both phages caused the primary condition and factor to withstand various environmental stresses for phage therapy in the aquaculture water environment (Tovar et al., 2000).

#### Bacterial Killing Assays

**FIGURE 3:**
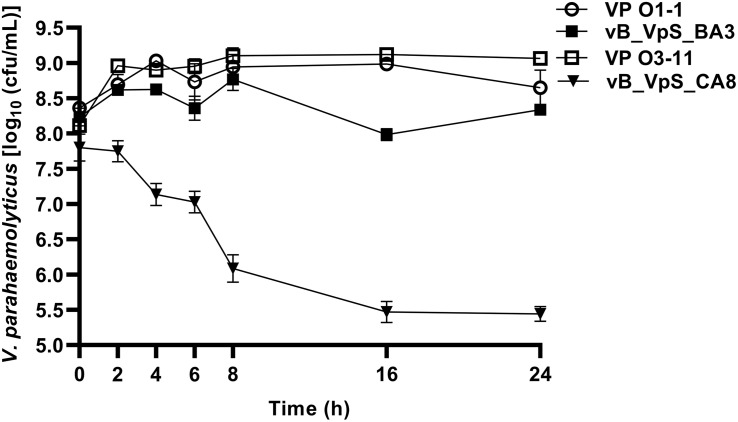
Bacterial growth reduction. Reduction in the exponential growth phase of VP O1-1 and VP O3-11 by phage BA3 and CA8 at an approximate MOI of 0.1. The given values are the means of three determinations.

To further assess potential applications, two phages were used for bacterial killing assays against hosts of *V. parahaemolyticus.* As shown in Figure 3, bacterial density in the bacterial control (BC, VP O1-1 and O3-11) increased by ∼1.00 log cfu/mL after 2 h of incubation. Compared to BC (VP O1-1), the treatment group (VP O1-1 + phage BA3) did not present significant differences before 16 h of incubation. However, the maximum inactivation with phage BA3 was 1.00 log cfu/mL, respectively, achieved at 16 h of incubation. During treatment, the rate of inactivation of phage CA8 was significantly higher than that obtained with phage BA3. Bacterial inactivation of phage CA8 was initiated after 2 h and the bacterial density was 1.21 log cfu/mL less than BC (VP O3-11). Moreover, maximum inactivation with phage CA8 was 3.65 and 3.63 log cfu/mL at 16 and 24 h of incubation. These data revealed that CA8 may have more potential than BA3 for biocontrol of multidrug-resistant *V. parahaemolyticus*.

### General Features of the Phage Genome

To further understand the general features of phages, the genome of phage BA3/CA8 was sequenced and analyzed. Mapping revealed that phage BA3 harbored a ∼1.0-kb fragment while phage CA8 harbored a ∼3.2-kb fragment with a ∼2-fold coverage; this revealed the termini considered terminal redundancy. Average frequency, the highest frequency, and ratio (R = highest frequency/average frequency) were calculated to verify the genome termini of both phages. Table 1 showed that BA3 (*R* = 54.88) and CA8 (*R* = 70.44) indicated the preferred termini. Our findings were similar to those of T4 like phages. Unlike the other phages with unique termini, however, functional proteins of both phages were directly predicted using the contig assembly.

**TABLE 1:** The criteria for distinguish termini.

**Phage**	**Number of reads**	**Gemone length (bp)**	**Highest frequency**	**Average frequency**	**Ratio**	**T1-F/T2-F**	**T1-R/T2-R**	**Characteristic**
vB_VpS_BA3	66260	58648	0.56	31	54.88	2.07	1.11	Preferred termini
vB_VpS_CA8	74722	58480	0.64	45	70.44	2.81	1.23	Preferred termini

The properties of the functional genome, including positions, directions, length, product, and putative functions of each gene are showed in Supplementary Tables S2, S3. Both phages had a linear, double-stranded DNA genome. The BA3 genome was 58,648 bp with a GC content of 46.30%, while that of CA8 was 58,480 bp with an average GC content of 46.42%. In total, 85 putative ORFs were predicted in the genome of phage BA3 (Supplementary Table S2), with average gene length of 628 bp, and sizes ranging from 126 to 2,529 nucleotides. In contrast, there were 84 putative ORFs in CA8 (Supplementary Table S3), with average gene length of 629 bp, and the same size range of nucleotides as BA3. Only 34 ORFs in both phage genomes were predicted and determined to be functional, and 51 in BA3 (50 in CA8) were assigned to hypothetical proteins based on the assumption that sequence homology reflects a functional relationship. Finally, no rRNA was detected, but only one tRNA gene (encoding Pro) was present in both phages. Therefore, the gene inventory of the two phages is largely uncharacterized. All data revealed that both phages had a similar genome size.

**FIGURE 4:**
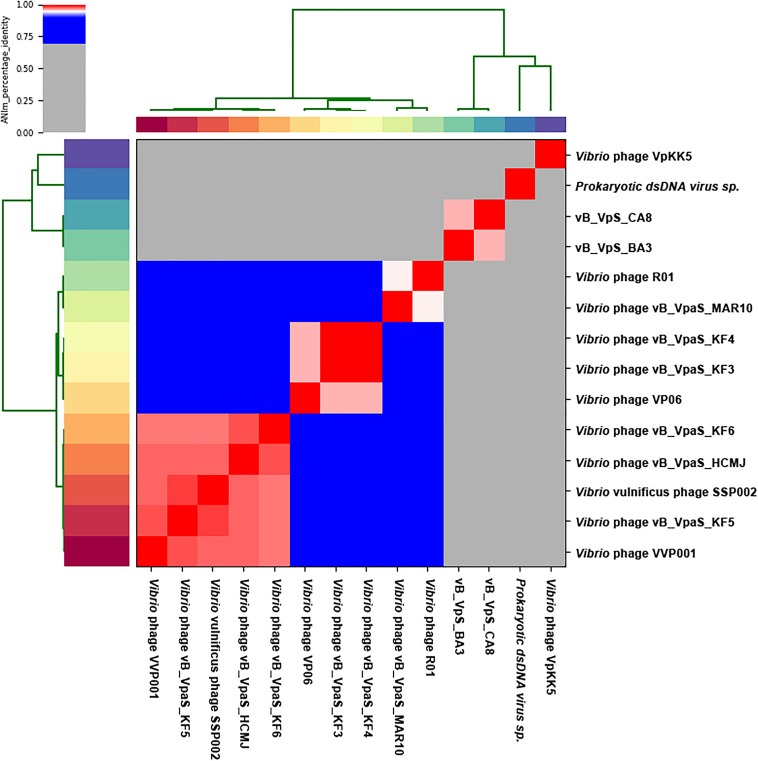
Heatmap of the average nucleotide identity (ANI) values for 14 whole genomes, sequenced from related phages of the family *Siphoviridae* in the NCBI database, including two phages from this study. Values range from 0 (0%) ANI to 1 (100% ANI): gray represents 0% ANI; clusters of highly similar phages are highlighted in pink and red.

The Bacterial and Archaeal Viruses Subcommittee (BAVS) described genus as a cohesive group of viruses sharing a high degree of nucleotide sequence similarity (>50%), with two viruses belonging to the same species differing by less than 5% from each other at the nucleotide level using BLASTn. In this study, phage BA3 had high identity (72.59 and 65.52%) to *Prokaryotic dsDNA virus* sp. (GenBank: MK892600.1) and *Vibrio* phage VpKK5 (GenBank: KM378617), with 14% coverage and 9% coverage, respectively. Phage CA8 had the same result in BLASTN with *Prokaryotic dsDNA virus* sp. Besides, it shared 65.25% identity with the *Vibrio* phage, VpKK5, and had 8% coverage. Phage CA8 however shared 69.45, 68.84, 68.84, 68.68, and 68.57% identity with the *Vibrio* phage VP06 (MG893203.1), *Vibrio* phage vB_VpaS_KF4 (MF754114.1), *Vibrio* phage vB_VpaS_KF3 (MF754113.1), *Vibrio* phage R01 (MH599087.1), and *Vibrio* phage VVP001 (MG602476.1), respectively, with 3% coverage. On analyzing the results of the heatmap (Figure 4) and regarding the ANIm percentage identity data (Supplementary Table S4), phage BA3 and phage CA8 displayed no similarity to any other bacteriophages used in the present ANI analysis. According to these data, we can conclude that both phages BA3 and CA8 had great difference relative to other phages in the NCBI database. Furthermore, we could predict that they are novel phages in the family, *Siphoviridae*. Lastly, nucleotide pairwise sequence alignment based on BLASTN revealed that phages BA3 and CA8 were 97.32% identical to each other while ANI was 96.48%; thus, they can be considered different isolates of the same phage species.

**FIGURE 5:**
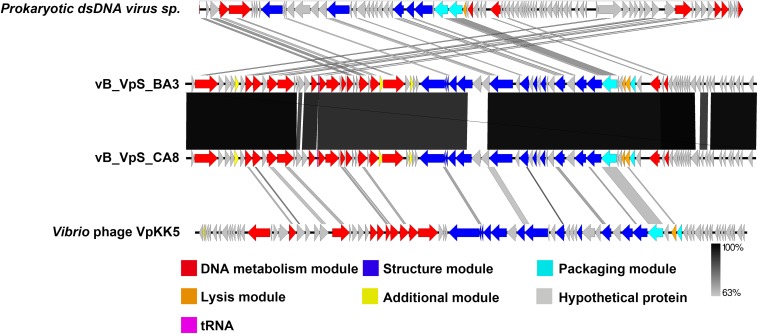
Schematic representation of the genomic organization of vB_VpS_BA3 and vB_VpS_CA8, compared to other related *Vibrio* phages in NCBI. The genome is divided into a DNA metabolism module (red), a lysis module (orange), a packaging module (cyan), a structure module (royal blue), additional functions (yellow), hypothetical proteins (gray), and tRNA (purple). Arrows indicate ORFs with either rightward or leftward direction.

The global genome comparison map among different phages is presented in Figure 5. All functional ORFs, displaying homology but low identity to amino acid sequences in the databases, were divided into four modules. Our findings proved that BA3 and CA8 were novel phages of *Siphoviridae*. Among the 34 genes with detected homologs, 94.1% appeared to be related to viruses while 5.9% had homologs in several groups of bacteria. These modules included DNA metabolism module, lysis module, packaging module, structure module, and additional module (Lal et al., 2016; Wong et al., 2019). In terms of phage therapy, lysis module and structure module (phage tail, receptor binding protein, RBP) played a direct role in bacterial lysis (Nobrega et al., 2018; Cahill and Young, 2019). However, *in silico* analysis of holin and *N*-acetylmuramoyl-L-alanine amidase revealed that phage BA3 and phage CA8 displayed no difference in the lytic mechanism and the ability at the end of the lytic cycle. Although both phages had a high degree of similarity in morphological characteristics and genomic features, the host range between them was not identical. Increasing research confirmed aspects of a process called adsorption between RBP and compatible cell surface structures (receptor) (Nobrega et al., 2018). For siphophages that infect gram-negative bacteria, this process was closely related to a central, straight fiber, as well as side fibers, but not a baseplate-like structure (Nobrega et al., 2018). In this study, any main fiber was either predicted or annotated, but there were five tailed assembly proteins (TAPs) in the genome of two phages. Notably, vB_VpS_BA3 gp35 shared 90.50% identity to vB_VpS_CA8 gp34 and vB_VpS_BA3 gp38 displayed 86.14% identity to vB_VpS_CA8 gp37. Meanwhile, vB_VpS_BA3 gp39 had the lowest identity (63.39%) to vB_VpS_CA8 gp38 among the comparative features of phage tail proteins. The other TAPs, tail length tape-measure protein and tail subunit in phage BA3 shared > 95.00% identity with these proteins in phage CA8. Such features indicated that the great differences in host range between the two phages were primarily caused by the low similarity of TAPs. Although the type II secretion system protein (BA3 gp6 and CA8 gp6) was detected and annotated, there were no core domains or related functions by InterPro. Moreover, no other virulent gene or factor was present, thus, we can conclude that both phages are safe for use in phage therapy for the prevention and control of *V. parahaemolyticus*.

**FIGURE 6:**
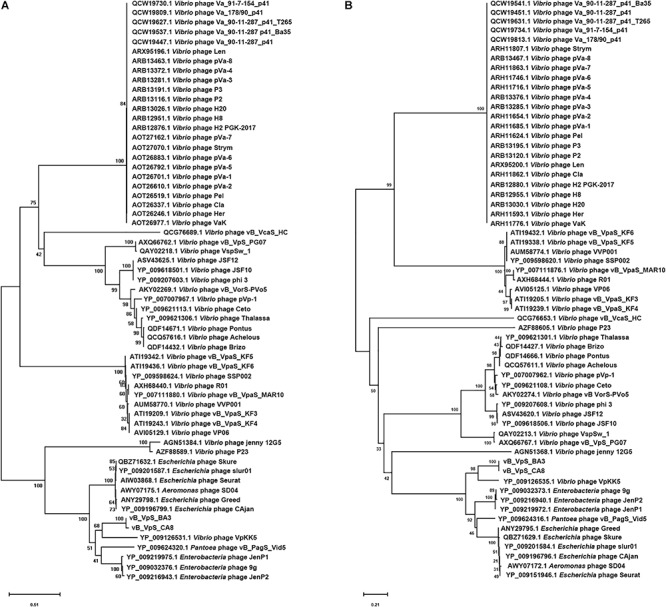
Phylogenetic tree showing vB_VpS_BA3, vB_VpS_CA8, and other related phages of the family *Siphoviridae* and the order *Caudovirales*. The amino acid sequences of **(A)** the major capsid protein and **(B)** the terminase large subunit of the related phages were downloaded from NCBI.

As a common practice, the phylogenetic tree was edited and visualized to analyze the evolution and identification of phage. The major capsid protein and terminase large subunit are conserved proteins in the bacteriophage genomes and regularly used to group phages in families by single gene analysis (Tetart et al., 2001; Fouts et al., 2013). In this study, proteomic comparison of available amino acid sequences of major capsid protein and terminase large subunit were considered to identify distant bacteriophage relatives via phylogenetic analysis. As shown in Figure 6, both the major capsid protein tree (Figure 6A) and terminase large subunit tree (Figure 6B) revealed that the phages BA3, CA8, and VpKK5, which are distinct from other siphophages in the NCBI database, could be divided into the same large cluster. Thus, it is suggested that they could originate from a common distant ancestor. However, phages BA3 and CA8 were still distinct from *Vibrio* phage VpKK5, which indicated that both isolates represented a novel *Vibrio* phage.

#### Analysis of Phage Virion Proteomes

*Siphoviridae* phage particle was mainly composed of structural proteins (structure module and packaging module) and a genomic DNA molecule inside its head. Genomic analysis predicted 12 genes in phage BA3 and CA8 genome coding proteins of structure module, including five TAPs, tail length tape-measure protein, tail subunit, virion structural protein, major head protein, minor head protein and structural phage protein. BlastP analysis showed there were terminase large subunit and terminase small subunit of packing module in both phages. All of these proteins were made up of whole phage scaffold. To identify the structural proteins in the mature phage BA3 and CA8, purified phage virions were analyzed by Nano LC-MS/MS.

Results showed that nine structural proteins were identified in phage BA3 virions (Supplementary Table S5), including two TAPs (gp38, gp39), tail length tape-measure protein, tail subunit, virion structural protein, major head protein, structural phage protein and terminase large subunit; 11 were identified in phage CA8 (Supplementary Table S6), including four TAPs (gp34, gp36, gp37, gp38), tail length tape-measure protein, tail subunit, virion structural protein, major head protein, minor head proteins and structural phage protein and terminase large subunit. Besides, annotated structural proteins in phage BA3 (gp35, gp36, gp37, gp46, gp53, and gp60) and phage CA8 (gp35, gp45, and gp59) were absent in our triplicate repeated proteomic analysis which may due to low copy number (Yuan and Gao, 2016). According to the differences of both host range and TAPs identified by LC-MS/MS between BA3 and CA8, it was considerate that research on transcriptomics and proteomics should be performed to further investigate the interaction of host-phage. Interestingly, DNA metabolism module related genes were also determined in our phage particles (BA3 gp2, gp8, gp9, gp12, gp17, gp18, gp19, gp23, gp16, gp28 and gp62; CA8 gp2, gp12 and gp22). It’s believed that the real function of these proteins might be involved in DNA translocation, transcription, repair, chromatin rearrangement repair, recombination and replication, which may play important roles in the phage early infection processes and will be our interests in the further studies as well (Lal et al., 2016; Yang M. et al., 2019).

## Conclusion

Numerous reports have recently warned that *V. parahaemolyticus* is a crucial cause of food poisoning that is associated with the consumption of seafood and ready-to-eat food. Not only is phage therapy a safe and pollution-free treatment, but it is regarded as a promising strategy and activity of biocontrol owing to its specific infectivity toward target *V. parahaemolyticus* and no direct negative effects on aquatic organisms and humans. In this work, we characterized and compared the interaction of vB_VpS_BA3 and vB_VpS_CA8 as novel members of *Siphoviridae* with their own host. Firstly, phage CA8 could infect over 40% *V. parahaemolyticus* isolates than phage BA3 when host range was determined. Secondly, although phage CA8 had a greater burst size than phage BA3, both phages were revealed to be promising candidates of biocontrol. Moreover, both phages with their short latent period could recognize and adsorb the target *V. parahaemolyticus* strains, indicating their ability to control and kill bacterial cells rapidly and effectively. In our further studies, phage therapy *in vitro* could be conducted to support the results. However, studies regarding potential applications of phages in animal and aquatic products are required to test and verify their safety. As such great number of ORFs could not be identified or characterized, this promoted us to determine the relevant genes and proteins for phage therapy. Because of the absence of virulent genes, both phages in our study were safe for use as phage therapy for the prevention and control of *V. parahaemolyticus*, ultimately stabilizing the progress of aquatic economy, enhancing food safety, and safeguarding human health.

## Data Availability Statement

All datasets generated for this study are included in the article/Supplementary Material.

## Author Contributions

JZ, QW, and MY conceived the study. MY and YL designed and conducted the experiments and wrote the manuscript. SH, JW, HC, XG, and YY contributed to the data analyze. QW and ZT guided the experiment. All authors agreed to be accountable for the content of the work.

## Conflict of Interest

The authors declare that the research was conducted in the absence of any commercial or financial relationships that could be construed as a potential conflict of interest.

## Footnotes

1 https://blast.ncbi.nlm.nih.gov/Blast.cgi
