# Supplementary material for: Isolation and Characterization of the Novel Phages vB_VpS_BA3 and vB_VpS_CA8 for Lysing Vibrio parahaemolyticus
Source: Front Microbiol. 2020 Feb 21;11:259. doi: 10.3389/fmicb.2020.00259 (PMC7047879; doi:10.3389/fmicb.2020.00259)
Supplement: Supplementary file 1 [file Data_Sheet_1.PDF]

Table. S1 Lytic spectra of vB_VpS_BA3 and vB_VpS_CA8 determined on 61 strains of *V. parahaemolyticus*

No.	*Vibrio parahaemolyticus* strain	Source	Location	Serotype	vB_VpS_CA8	vB_VpS_BA3	Antibiotic resistance
1	O1-1	Shrimp	Hangzhou	O1	-	+	CN-K-CIP-S-AMP
2	O1-2	Shrimp	Taiyuan	O1	-	-	CN-K-S-AMP
3	O1-3	Shrimp	Shenzhen	O1	-	+	K-CIP-S-AMP-C
4	O1-4	Cooked chicken	Heyuan	O1	-	-	K-S-AMP
5	O2-5	Shrimp	Xian	O2	+	-	S-AMP
6	O2-6	Fish	Shenzhen	O2	-	+	S-AMP
7	O2-7	Shrimp	Heyuan	O2	+	-	CIP-S-AMP
8	O3-8	Shrimp	Panyu	O3	-	-	K-CIP-S-AMP
9	O3-9	Fish	Shaoguan	O3	+	-	CN-K-CIP-S-AMP
10	O3-10	Shrimp	Beijing	O3	+	-	-
11	O3-11	Shrimp	Zengcheng	O3	+	-	CN-S-AMP-C
12	O4-12	Cooked chicken	Xiamen	O4	-	-	CIP-S-AMP
13	O4-13	Fish	Shaoguan	O4	-	-	S-AMP
14	O4-14	Fish	Shaoguan	O4	-	-	AMP
15	O5-15	Shrimp	Panyu	O5	+	-	CN-K-CIP-S-AMP
16	O5-16	Salad	Conghua	O5	-	-	-
17	O5-17	Shrimp	Yuexiu	O5	-	-	K-S-AMP
18	O6-18	Shrimp	Zhangjiang	O6	-	-	K-CIP-S-AMP
19	O6-19	Shrimp	Shaoguan	O6	-	-	CIP-AMP
20	O6-20	Shrimp	Nanjing	O6	-	-	CIP-S-AMP
21	O8-21	Fish	Jinan	O8	-	-	S-AMP
22	O8-22	Fish	Conghua	O8	-	-	-
23	O8-23	Shrimp	Conghua	O8	-	-	-
24	O9-24	Fish	Haikou	O9	+	-	CN-CIP-S-AMP
25	O10-25	Fish	Zhangjiang	O10	-	-	S-AMP
26	O10-26	Shrimp	Nanning	O10	-	-	CN-CIP-S-AMP-C
27	O10-27	Shrimp	Beijing	O10	-	-	CN-S-AMP
28	O10-28	Shrimp	Conghua	O10	+	-	AMP
29	O11-29	Shrimp	Lanzhou	O11	+	-	CN-K-CIP-AMP
30	O11-30	Shrimp	Shenyang	O11	-	-	S-AMP

31	O11-31	Shrimp	Lanzhou	O11	-	-	CIP-S-AMP
32	O12-32	Shrimp	Panyu	O12	+	-	S-AMP
33	O12-33	Fish	Beihai	O12	-	-	CIP-S-AMP
34	O12-34	Shrimp	Beihai	O12	+	-	SXT-CIP-AMP-TE-K
35	O12-35	Shrimp	Shijiazhuang	O12	+	-	CN-CIP-S-AMP
36	O1-36	Shrimp	Yuexiu	O1	-	-	K-CIP-S-AMP
37	O1-37	Fish	Panyu	O1	+	-	CN-CIP-S-AMP
38	O1-38	Fish	Panyu	O1	-	-	CIP-S-AMP
39	O2-39	Shrimp	Panyu	O2	+	-	K-CIP-S-AMP-C
40	O2-40	Shrimp	Yuexiu	O2	+	-	S-AMP
41	O2-41	Shrimp	Yuexiu	O2	-	-	CIP-AMP
42	O3-42	Shrimp	Zengcheng	O3	-	+	K-CIP-S-AMP
43	O3-43	Fish	Panyu	O3	-	-	S-AMP
44	O3-44	Shrimp	Panyu	O3	-	-	CN-K-CIP-S-AMP-C
45	O4-45	Shrimp	Conghua	O4	+	-	K-S-AMP
46	O4-46	Shrimp	Zengcheng	O4	+	-	K-CIP-S-AMP
47	O4-47	Cooked pork	Conghua	O4	-	-	CIP-S-AMP
48	O4-48	Shrimp	Panyu	O4	-	-	CIP-AMP
49	O5-49	Cooked pork	Zhangjiang	O5	+	-	CIP-S-AMP
50	O5-50	Shrimp	Panyu	O5	-	+	CN-K-CIP-S-AMP
51	O8-51	Fish	Conghua	O8	-	-	CN-K-CIP-S-C
52	O8-52	Shrimp	Yuexiu	O8	+	-	K-CIP-S-AMP
53	O8-53	Fish	Conghua	O8	-	-	S-AMP
54	O10-54	Shrimp	Zhangjiang	O10	+	-	K-CIP-S-AMP
55	O10-55	Shrimp	Zengcheng	O10	+	-	CN-K-CIP-S-AMP-C
56	O11-56	Shrimp	Yuexiu	O11	+	-	CIP-S-AMP
57	O11-57	Shrimp	Zengcheng	O11	-	-	S-AMP
58	O11-58	Shrimp	Zengcheng	O11	-	-	S-AMP-C
59	O12-59	Fish	Liwan	O12	-	-	K-CIP-S-AMP-C
60	O12-60	Shrimp	Conghua	O12	-	-	CN-K-CIP-S-AMP-C
61	O12-61	Shrimp	Panyu	O12	-	-	CN-K-CIP-S-AMP

CN: Centamicin, K: Kanamycin, CIP: Ciprofloxacin, S: Streptomycin, AMP: Ampicillin, C: Chloramphenicol.

Clear lysis zone (+), no lysis zone (−).

Table. S2 Functional classification of 85 ORFs in the vB_VpS_BA3 genome

Label	Strand	Start	Stop	Length（nt | aa）	Product	Function protein	Accession	Query Cover	E value	Identidy
vB_VpS_BA3 gp1	+	677	994	318 | 105	hypothetical protein	[*Verrucomicrobiales bacterium*]	MBG89565.1	57.00%	5.00E-10	42.62%
vB_VpS_BA3 gp2	+	999	3380	2382 | 793	primase	[*Pantoea* phage vB_PagS_Vid5]	YP_009624375.1	96.00%	3.00E-86	27.96%
vB_VpS_BA3 gp3	+	3451	3975	525 | 174	hypothetical protein	[*Vibrio* phage 1.251.O._10N.261.55.E5]	AUR98471.1	91.00%	1.00E-14	32.53%
vB_VpS_BA3 gp4	+	4120	4695	576 | 191	hypothetical protein	[*Escherichia coli*]	WP_038977048.1	80.00%	5.00E-10	31.68%
vB_VpS_BA3 gp5	+	4685	5116	432 | 143	hypothetical protein	[*Pseudomonas* phage PaMx25]	YP_009603606.1	83.00%	1.00E-11	34.71%
vB_VpS_BA3 gp6	+	5106	5516	411 | 136	type II secretion system protein	[*Grimontia hollisae*]	WP_005502247.1	97.00%	3.00E-11	36.62%
vB_VpS_BA3 gp7	+	5705	6139	435 | 144	hypothetical protein	[*Edwardsiella* phage KF-1]	YP_006990453.1	100.00%	4.00E-10	32.64%
vB_VpS_BA3 gp8	+	6190	6933	744 | 247	ATPase	[*Vibrio* phage VpKK5]	YP_009126576.1	98.00%	1.00E-96	56.97%
vB_VpS_BA3 gp9	+	6946	7869	924 | 307	multimodular transpeptidase-transglycosylase	[*Vibrio* phage vB_VhaS-tm]	ANO57479.1	43.00%	7.00E-52	62.69%
vB_VpS_BA3 gp10	+	7951	8424	474 | 157	hypothetical protein	[*Stenotrophomonas* phage vB_SmaS-DLP_1]	AKI28809.1	92.00%	5.00E-23	35.33%
vB_VpS_BA3 gp11	+	8418	9494	1077 | 358	exonuclease	[*Pantoea* phage vB_PagS_Vid5]	YP_009624360.1	82.00%	2.00E-79	42.19%
vB_VpS_BA3 gp12	+	9484	11196	1713 | 570	DNA/RNA helicase	[*Vibrio* phage VpKK5]	YP_009126858.1	97.00%	0.00E+00	53.08%
vB_VpS_BA3 gp13	+	11159	11371	213 | 70	hypothetical protein	[*Vibrio* phage VpKK5]	YP_009126859.1	98.00%	2.00E-09	43.48%
vB_VpS_BA3 gp14	+	11438	11653	216 | 71	hypothetical protein	-	-	-	-	-

vB_VpS_BA3 gp15	+	11658	12236	579 | 192	hypothetical protein	[*Vibrio* phage Ares1]	AUR81191.1	25.00%	3.00E-13	63.27%
vB_VpS_BA3 gp16	+	12236	12820	585 | 194	hypothetical protein	[*Pseudomonas* phage PaMx25]	YP_009603593.1	32.00%	1.00E-11	52.38%
vB_VpS_BA3 gp17	+	12938	13594	657 | 218	transcriptional activator	[*Pantoea* phage vB_PagS_Vid5]	YP_009624356.1	32.00%	6.00E-20	54.29%
vB_VpS_BA3 gp18	+	13779	14483	705 | 234	orgamic radical activating enzyme	[*Vibrio* phage VpKK5]	YP_009126587.1	99.00%	9.00E-62	46.44%
vB_VpS_BA3 gp19	+	14490	15878	1389 | 462	putative QueC-like protein	[*Pseudomonas* phage PaMx25]	ALH23790.1	100.00%	3.00E-165	55.00%
vB_VpS_BA3 gp20	+	15859	16038	180 | 59	hypothetical protein	-	-	-	-	-
vB_VpS_BA3 gp21	+	16038	16502	465 | 154	6-pyruvoyl tetrahydropterin synthase	[*Vibrio* phage VpKK5]	YP_009126589.1	98.00%	4.00E-58	56.60%
vB_VpS_BA3 gp22	+	16475	16600	126 | 41	hypothetical protein	-	-	-	-	-
vB_VpS_BA3 gp23	+	16602	17225	624 | 207	type 1 GTP cyclohydrolase	[*Pantoea* phage vB_PagS_Vid5]	YP_009624349.1	92.00%	1.00E-87	68.59%
vB_VpS_BA3 gp24	+	17225	17473	249 | 82	hypothetical protein	[*Vibrio* phage SIO-2]	YP_004957517.1	96.00%	6.00E-10	36.71%
vB_VpS_BA3 gp25	+	17538	17840	303 | 100	hypothetical protein	-	-	-	-	-
vB_VpS_BA3 gp26	+	17842	18792	951 | 316	tRNA ribosyltransferase	[*Pantoea* phage vB_PagS_Vid5]	YP_009624348.1	98.00%	3.00E-143	64.10%
vB_VpS_BA3 gp27	+	18792	19004	213 | 70	hypothetical protein	-	-	-	-	-
vB_VpS_BA3 gp28	+	18997	19971	975 | 324	DNA polymerase beta subunit	[*Vibrio* phage VpKK5]	YP_009126592.1	98.00%	2.00E-99	44.97%
vB_VpS_BA3 gp29	+	19971	20324	354 | 117	DUF2493 domain-containing protein	[*Bradyrhizobium japonicum*]	WP_080587050.1	93.00%	2.00E-18	43.12%
vB_VpS_BA3	+	20314	22521	2208 | 735	putative DNA	[*Pseudomonas* phage	ALH23797.1	99.00%	0.00E+00	45.44%

gp30					polymerase	PaMx25]				
vB_VpS_BA3 gp31	+	22591	23100	510 | 169	hypothetical protein	-	-	-	-	-
vB_VpS_BA3 gp32	+	23100	23396	297 | 98	TM2 domain containing protein	[*Haliangium ochraceum* DSM 14365]	ACY17426.1	97.00%	4.00E-09	37.11%
vB_VpS_BA3 gp33	+	23380	23721	342 | 113	hypothetical protein	-	-	-	-	-
vB_VpS_BA3 gp34	+	23703	23990	288 | 95	hypothetical protein	[*Vibrio* phage vB_VhaS-tm]	ANO57467.1	96.00%	2.00E-29	50.00%
vB_VpS_BA3 gp35	-	26623	24095	2529 | 842	tail assembly protein	[*Vibrio* phage VpKK5]	YP_009126597.1	93.00%	9.00E-172	40.08%
vB_VpS_BA3 gp36	-	26793	26596	198 | 65	tail assembly protein	[*Vibrio* phage VpKK5]	YP_009126598.1	100.00%	8.00E-27	69.23%
vB_VpS_BA3 gp37	-	27010	26795	216 | 71	tail assembly protein	[*Vibrio* phage VpKK5]	YP_009126599.1	100.00%	2.00E-19	55.56%
vB_VpS_BA3 gp38	-	27823	27020	804 | 267	tail assembly protein	[*Vibrio* phage VpKK5]	YP_009126600.1	94.00%	1.00E-61	41.18%
vB_VpS_BA3 gp39	-	29460	27823	1638 | 545	tail assembly protein	[*Vibrio* phage VpKK5]	YP_009126601.1	99.00%	2.00E-69	29.22%
vB_VpS_BA3 gp40	-	30386	29463	924 | 307	hypothetical protein	[*Vibrio* phage vB_VpaS_MAR10]	YP_007111891.1	91.00%	3.00E-51	34.98%
vB_VpS_BA3 gp41	-	31210	30386	825 | 274	hypothetical protein	[*Vibrio* phage vB_VpaS_MAR10]	YP_007111890.1	100.00%	2.00E-93	52.73%
vB_VpS_BA3 gp42	-	33612	31210	2403 | 800	tail length tape-measure protein 1	[*Vibrio* phage vB_VhaS-tm]	ANO57505.1	100.00%	0.00E+00	49.45%
vB_VpS_BA3 gp43	-	34299	33892	408 | 135	hypothetical protein	[*Vibrio* phage vB_VhaS-tm]	WP_079791462.1	96.00%	4.00E-20	37.69%
vB_VpS_BA3 gp44	-	35260	34310	951 | 316	tail subunit	[*Vibrio* phage VpKK5]	YP_009126524.1	100.00%	2.00E-115	55.14%
vB_VpS_BA3 gp45	-	35681	35262	420 | 139	hypothetical protein	[*Vibrio* phage vB_VhaS-tm]	ANO57510.1	97.00%	8.00E-09	31.16%
vB_VpS_BA3	-	36097	35678	420 | 139	virion structural protein	[*Pseudomonas* phage	YP_009210635.1	94.00%	1.00E-18	35.11%

gp46						PaMx28]				
vB_VpS_BA3 gp47	-	36452	36075	378 | 125	hypothetical protein	[*Vibrio* phage VpKK5]	YP_009126527.1	96.00%	4.00E-07	33.88%
vB_VpS_BA3 gp48	-	36976	36449	528 | 175	virion structural protein	[*Pseudomonas* phage PaMx25]	ALH23816.1	100.00%	3.00E-16	36.67%
vB_VpS_BA3 gp49		37211	37023	189 | 62	hypothetical protein	-	-	-	-	-
vB_VpS_BA3 gp50	-	37862	37224	639 | 212	hypothetical protein	[*Vibrio* phage VpKK5]	YP_009126530.1	36.00%	1.00E-12	44.16%
vB_VpS_BA3 gp51	-	38964	37906	1059 | 352	major head protein	[*Pseudomonas* phage PaMx25]	ALH23820.1	95.00%	2.00E-147	60.53%
vB_VpS_BA3 gp52	-	39967	39098	870 | 289	hypothetical protein	[*Vibrio* phage vB_VhaS-tm]	ANO57523.1	98.00%	8.00E-75	48.98%
vB_VpS_BA3 gp53	-	41157	39994	1164 | 387	minor head protein	[*Vibrio* phage VpKK5]	YP_009126533.1	99.00%	2.00E-58	30.51%
vB_VpS_BA3 gp54	-	42724	41144	1581 | 526	structural phage protein	[*Vibrio* phage VpKK5]	YP_009126534.1	94.00%	3.00E-122	44.38%
vB_VpS_BA3 gp55	-	44302	42734	1569 | 522	terminase large subunit	[*Vibrio* phage VpKK5]	YP_009126535.1	97.00%	0.00E+00	69.73%
vB_VpS_BA3 gp56	-	44480	44268	213 | 70	hypothetical protein	-	-	-	-	-
vB_VpS_BA3 gp57	-	44841	44431	411 | 136	hypothetical protein	-	-	-	-	-
vB_VpS_BA3 gp58	-	45151	44819	333 | 110	holin	[*Salmonella* phage PMBT28]	AUZ95557.1	82.00%	2.00E-19	42.86%
vB_VpS_BA3 gp59	-	45642	45151	492 | 163	N-acetylmuramoyl-L-ala nine amidase	[*Vibrio* phage VpKK5]	YP_009126538.1	98.00%	2.00E-51	53.09%
vB_VpS_BA3 gp60	-	46175	45639	537 | 178	terminase small subunit	*[Escherichia* phage Greed]	ANY29794.1	98.00%	3.00E-25	36.31%
vB_VpS_BA3 gp61	-	46784	46278	507 | 168	hypothetical protein	[*Vibrio* phage vB_VhaS-tm]	ANO57533.1	42.00%	1.00E-18	51.39%
vB_VpS_BA3	-	48711	47650	1062 | 353	DNA ligase	[*Vibrio* phage VPMS1]	YP_008239685.1	85.00%	5.00E-18	29.30%

gp62										
vB_VpS_BA3 gp63	-	49094	48807	288 | 95	hypothetical protein	[*Pandoraea pnomenusa*]	WP_052240415.1	85.00%	2.00E-04	33.33%
vB_VpS_BA3 gp64	-	49519	49091	429 | 142	endonuclease	[*Pantoea* phage vB_PagS_Vid5]	AVJ51809.1	90.00%	4.00E-26	45.52%
vB_VpS_BA3 gp65	-	49938	49519	420 | 139	hypothetical protein	[*Salmonella* phage Maynard]	YP_008771018.1	93.00%	2.00E-09	30.71%
vB_VpS_BA3 gp66	-	50389	49928	462 | 153	hypothetical protein	-	-	-	-	-
vB_VpS_BA3 gp67	-	50587	50393	195 | 64	hypothetical protein	[*Vibrio* phage vB_VhaS-tm]	ANO57458.1	89.00%	3.00E-07	46.67%
vB_VpS_BA3 gp68	-	50843	50595	249 | 82	hypothetical protein	[*Vibrio* phage vB_VhaS-tm]	ANO57460.1	91.00%	3.00E-28	65.33%
vB_VpS_BA3 gp69	-	51031	50843	189 | 62	hypothetical protein	[*Vibrio* phage vB_VhaS-tm]	ANO57459.1	93.00%	1.00E-26	77.59%
vB_VpS_BA3 gp70	-	51303	51028	276 | 91	hypothetical protein	-	-	-	-	-
vB_VpS_BA3 gp71	-	51547	51287	261 | 86	hypothetical protein	-	-	-	-	-
vB_VpS_BA3 gp72	-	51776	51522	255 | 84	hypothetical protein	-	-	-	-	-
vB_VpS_BA3 gp73	-	52390	51776	615 | 204	hypothetical protein	-	-	-	-	-
vB_VpS_BA3 gp74	-	52845	52390	456 | 151	hypothetical protein	[*Vibrio* phage VPMS1]	YP_008239705.1	98.00%	2.00E-44	47.97%
vB_VpS_BA3 gp75	-	53212	52919	294 | 97	hypothetical protein	-	-	-	-	-
vB_VpS_BA3 gp76	-	53629	53375	255 | 84	hypothetical protein	-	-	-	-	-
vB_VpS_BA3 gp77	-	53944	53639	306 | 101	hypothetical protein	-	-	-	-	-
vB_VpS_BA3	-	54315	53938	378 | 125	hypothetical protein	-	-	-	-	-

gp78										
vB_VpS_BA3 gp79	-	55115	54819	297 | 98	hypothetical protein	-	-	-	-	-
vB_VpS_BA3 gp80	-	55503	55207	297 | 98	hypothetical protein	-	-	-	-	-
vB_VpS_BA3 gp81	-	56089	55757	333 | 110	hypothetical protein	-	-	-	-	-
vB_VpS_BA3 gp82	-	56322	56101	222 | 73	hypothetical protein	-	-	-	-	-
vB_VpS_BA3 gp83	-	56896	56438	459 | 152	hypothetical protein	[*Pseudomonas* phage PaMx25]	ALH23785.1	94.00%	1.00E-21	41.61%
vB_VpS_BA3 gp84	-	57243	56974	270 | 89	hypothetical protein	-	-	-	-	-
vB_VpS_BA3 gp85	-	58270	57785	486 | 161	hypothetical protein	-	-	-	-	-

Table. S3 Functional classification of 84 ORFs in the vB_VpS_CA8 genome

Label	Strand	Start	Stop	Length(nt | aa)	Pruduct	Function protein	Accession	Query Cover	E value	Identidy
vB_VpS_CA8 gp1	+	550	867	318 | 105	hypothetical protein	[*Vibrio* phage vB_VhaS-tm]	ANO57473.1	57.00%	1.00E-04	37.70%
vB_VpS_CA8 gp2	+	872	3253	2382 | 793	primase	[*Pantoea* phage vB_PagS_Vid5]	YP_009624375.1	96.00%	1.00E-84	27.36%
vB_VpS_CA8 gp3	+	3324	3848	525 | 174	hypothetical protein	[*Vibrio* phage 1.251.O._10N.261.55.E5]	AUR98471.1	91.00%	1.00E-14	32.53%
vB_VpS_CA8 gp4	+	3993	4568	576 | 191	hypothetical protein	[*Escherichia coli*]	WP_038977048.1	80.00%	6.00E-10	31.68%
vB_VpS_CA8 gp5	+	4558	4989	432 | 143	hypothetical protein	[*Pseudomonas* phage PaMx25]	YP_009603606.1	83.00%	1.00E-11	34.71%
vB_VpS_CA8 gp6	+	4979	5389	411 | 136	type II secretion system protein	[*Grimontia hollisae*]	WP_005502247.1	97.00%	3.00E-11	36.62%
vB_VpS_CA8 gp7	+	5578	6012	435 | 144	hypothetical protein	[*Edwardsiella* phage KF-1]	YP_006990453.1	100.00%	3.00E-10	32.64%
vB_VpS_CA8 gp8	+	6063	6806	744 | 247	ATPase	[*Vibrio* phage VpKK5]	YP_009126576.1	98.00%	1.00E-96	56.97%
vB_VpS_CA8 gp9	+	6819	7742	924 | 307	multimodular transpeptidase-transglycosy lase	[*Vibrio* phage vB_VhaS-tm]	ANO57479.1	43.00%	6.00E-52	62.69%
vB_VpS_CA8 gp10	+	7825	8298	474 | 157	hypothetical protein	[*Stenotrophomonas* phage vB_SmaS-DLP_1]	AKI28809.1	92.00%	2.00E-22	34.67%
vB_VpS_CA8 gp11	+	8292	9368	1077 | 358	exonuclease	[*Pantoea* phage vB_PagS_Vid5]	YP_009624360.1	82.00%	1.00E-79	42.19%
vB_VpS_CA8 gp12	+	9358	11067	1710 | 569	DNA/RNA helicase	[*Vibrio* phage VpKK5]	YP_009126858.1	97.00%	0.00E+00	52.55%
vB_VpS_CA8 gp13	+	11030	11242	213 | 70	hypothetical protein	[*Vibrio* phage VpKK5]	YP_009126859.1	98.00%	2.00E-09	43.48%
vB_VpS_CA8 gp14	+	11309	11902	594 | 197	hypothetical protein	[*Vibrio* phage Ares1]	AUR81191.1	53.00%	3.00E-12	39.05%
vB_VpS_CA8	+	11902	12486	585 | 194	hypothetical protein	[*Pseudomonas* phage		32.00%	1.00E-11	52.38%

gp15						PaMx25]	YP_009603593.1			
vB_VpS_CA8 gp16	+	12605	13276	672 | 223	transcriptional activator	[*Pantoea* phage vB_PagS_Vid5]	YP_009624356.1	31.00%	6.00E-20	54.29%
vB_VpS_CA8 gp17	+	13594	14298	705| 234	orgamic radical activating enzyme	[*Vibrio* phage VpKK5]	YP_009126587.1	99.00%	1.00E-61	46.44%
vB_VpS_CA8 gp18	+	14305	15693	1389 | 462	putative QueC-like protein	[*Pseudomonas* phage PaMx25]	ALH23790.1	100.00%	4.00E-165	55.00%
vB_VpS_CA8 gp19	+	15674	15853	180 | 59	hypothetical protein	-	-	-	-	-
vB_VpS_CA8 gp20	+	15853	16317	465 | 154	6-pyruvoyl tetrahydropterin synthase	[*Vibrio* phage VpKK5]	YP_009126589.1	98.00%	1.00E-57	56.44%
vB_VpS_CA8 gp21	+	16290	16415	126 | 41	hypothetical protein	-	-	-	-	-
vB_VpS_CA8 gp22	+	16417	17040	624 | 207	type 1 GTP cyclohydrolase	[*Pantoea* phage vB_PagS_Vid5]	YP_009624349.1	92.00%	1.00E-87	68.59%
vB_VpS_CA8 gp23	+	17040	17288	249 | 82	hypothetical protein	[*Vibrio* phage SIO-2]	YP_004957517.1	96.00%	6.00E-10	36.71%
vB_VpS_CA8 gp24	+	17353	17655	303 | 100	hypothetical protein	-	-	-	-	-
vB_VpS_CA8 gp25	+	17657	18607	951 | 316	tRNA ribosyltransferase	[*Pantoea* phage vB_PagS_Vid5]	YP_009624348.1	98.00%	3.00E-143	64.10%
vB_VpS_CA8 gp26	+	18607	18819	213 | 70	hypothetical protein	-	-			
vB_VpS_CA8 gp27	+	18812	19786	975 | 324	DNA polymerase beta subunit	[*Vibrio* phage VpKK5]	YP_009126592.1	98.00%	2.00E-99	44.97%
vB_VpS_CA8 gp28	+	19786	20139	354 | 117	DUF2493 domain-containing protein	[*Bradyrhizobium japonicum*]	WP_080587050.1	93.00%	6.00E-19	44.04%
vB_VpS_CA8 gp29	+	20129	22336	2208 | 735	putative DNA polymerase	[*Pseudomonas* phage PaMx25]	ALH23797.1	99.00%	0.00E+00	45.58%
vB_VpS_CA8 gp30	+	22594	22914	321 | 106	hypothetical protein	-	-	-	-	-
vB_VpS_CA8	+	22914	23210	297 | 98	TM2 domain containing	[*Haliangium ochraceum*	ACY17426.1	97.00%	5.00E-09	39.18%

gp31					protein	DSM 14365]				
vB_VpS_CA8 gp32	+	23194	23535	342 | 113	hypothetical protein	-	-	-	-	-
vB_VpS_CA8 gp33	+	23517	23804	288 | 95	hypothetical protein	[*Vibrio* phage vB_VhaS-tm]	ANO57467.1	96.00%	9.00E-29	48.96%
vB_VpS_CA8 gp34	-	26437	23909	2529 | 842	tail assembly protein	[*Vibrio* phage VpKK5]	YP_009126597.1	89.00%	1.00E-168	41.09%
vB_VpS_CA8 gp35	-	26607	26410	198 | 65	tail assembly protein	[*Vibrio* phage VpKK5]	YP_009126598.1	100.00%	4.00E-27	69.23%
vB_VpS_CA8 gp36	-	26824	26609	216 | 71	tail assembly protein	[*Vibrio* phage VpKK5]	YP_009126599.1	100.00%	5.00E-19	54.17%
vB_VpS_CA8 gp37	-	27636	26833	804 | 267	tail assembly protein	[*Vibrio* phage VpKK5]	YP_009126600.1	94.00%	9.00E-57	39.92%
vB_VpS_CA8 gp38	-	29279	27633	1647 | 548	tail assembly protein	[*Vibrio* phage VpKK5]	YP_009126601.1	99.00%	1.00E-74	29.89%
vB_VpS_CA8 gp39	-	30241	29282	960 | 319	hypothetical protein	[*Vibrio* phage VP06]	AVI05140.1	100.00%	2.00E-153	66.56%
vB_VpS_CA8 gp40	-	31074	30241	834 | 277	hypothetical protein	[*Vibrio* phage VP06]	AVI05139.1	99.00%	2.00E-176	82.25%
vB_VpS_CA8 gp41	-	33473	31074	2400 | 799	tail length tape-measure protein	[*Vibrio* phage vB_VhaS-tm]	ANO57505.1	100.00%	0.00E+00	49.88%
vB_VpS_CA8 gp42	-	34160	33753	408 | 135	hypothetical protein	[*Vibrio* phage vB_VhaS-tm]	WP_079791462.1	96.00%	4.00E-19	37.69%
vB_VpS_CA8 gp43	-	35121	34171	951 | 316	tail subunit	[*Vibrio* phage VpKK5]	YP_009126524.1	100.00%	4.00E-116	55.45%
vB_VpS_CA8 gp44	-	35542	35123	420 | 139	hypothetical protein	-	-	-	-	-
vB_VpS_CA8 gp45	-	35958	35539	420 | 139	virion structural protein	[*Pseudomonas* phage PaMx28]	YP_009210635.1	97.00%	3.00E-20	33.82%
vB_VpS_CA8 gp46	-	36313	35936	378 | 125	hypothetical protein	[*Vibrio* phage VpKK5]	YP_009126527.1	96.00%	4.00E-07	33.88%
vB_VpS_CA8	-	36837	36310	528 | 175	virion structural protein	[*Pseudomonas* phage	ALH23816.1	100.00%	5.00E-16	36.11%

gp47						PaMx25]				
vB_VpS_CA8 gp48	-	37072	36884	189 | 62	hypothetical protein	-	-	-	-	-
vB_VpS_CA8 gp49	-	37723	37085	639 | 212	hypothetical protein	[*Vibrio* phage VpKK5]	YP_009126530.1	36.00%	3.00E-11	42.86%
vB_VpS_CA8 gp50	-	38825	37767	1059 | 352	major head protein	[*Pseudomonas* phage PaMx25]	ALH23820.1	95.00%	2.00E-149	61.40%
vB_VpS_CA8 gp51	-	39828	38959	870 | 289	hypothetical protein	[*Vibrio* phage vB_VhaS-tm]	ANO57523.1	98.00%	2.00E-75	49.32%
vB_VpS_CA8 gp52	-	41018	39855	1164 | 387	minor head protein	[*Vibrio* phage VpKK5]	YP_009126533.1	99.00%	7.00E-59	30.51%
vB_VpS_CA8 gp53	-	42585	41005	1581 | 526	structural phage protein	[*Vibrio* phage VpKK5]	YP_009126534.1	94.00%	3.00E-122	44.38%
vB_VpS_CA8 gp54	-	44163	42595	1569 | 522	terminase large subunit	[*Vibrio* phage VpKK5]	YP_009126535.1	98.00%	0.00E+00	69.73%
vB_VpS_CA8 gp55	-	44341	44129	213 | 70	hypothetical protein	-	-	-	-	-
vB_VpS_CA8 gp56	-	44702	44292	411 | 136	hypothetical protein	-	-	-	-	-
vB_VpS_CA8 gp57	-	45012	44680	333 | 110	holin	[*Salmonella* phage PMBT28]	AUZ95557.1	82.00%	2.00E-19	42.86%
vB_VpS_CA8 gp58	-	45503	45012	492 | 163	N-acetylmuramoyl-L-alanine amidase	[*Vibrio* phage VpKK5]	YP_009126538.1	98.00%	1.00E-51	53.09%
vB_VpS_CA8 gp59	-	46036	45500	537 | 178	terminase small subunit	[*Escherichia* phage Greed]	ANY29794.1	98.00%	3.00E-25	36.31%
vB_VpS_CA8 gp60	-	46645	46139	507 | 168	hypothetical protein	[*Vibrio* phage vB_VhaS-tm]	ANO57533.1	42.00%	4.00E-15	48.61%
vB_VpS_CA8 gp61	-	48573	47512	1062 | 353	DNA ligase	[*Vibrio* phage VPMS1]	YP_008239685.1	85.00%	6.00E-18	29.30%
vB_VpS_CA8 gp62	-	49086	48799	288 | 95	hypothetical protein	[*Pandoraea pnomenusa*]	WP_052240415.1	85.00%	2.00E-04	33.33%
vB_VpS_CA8	-	49511	49083	429 | 142	endonuclease	[*Pantoea* phage	AVJ51809.1	90.00%	4.00E-26	45.52%

gp63						vB_PagS_Vid5]				
vB_VpS_CA8 gp64	-	49930	49511	420 | 139	hypothetical protein	[*Salmonella* phage Marshall]	YP_008771812.1	93.00%	3.00E-18	30.71%
vB_VpS_CA8 gp65	-	50381	49920	462 | 153	hypothetical protein	-	-	-	-	-
vB_VpS_CA8 gp66	-	50579	50385	195 | 64	hypothetical protein	[*Vibrio* phage vB_VhaS-tm]	ANO57458.1	89.00%	3.00E-07	46.67%
vB_VpS_CA8 gp67	-	50835	50587	249 | 82	hypothetical protein	[*Vibrio* phage vB_VhaS-tm]	ANO57460.1	91.00%	2.00E-28	65.33%
vB_VpS_CA8 gp68	-	51023	50835	189 | 62	hypothetical protein	[*Vibrio* phage vB_VhaS-tm]	ANO57459.1	93.00%	1.00E-26	77.59%
vB_VpS_CA8 gp69	-	51295	51020	276 |91	hypothetical protein	-	-	-	-	-
vB_VpS_CA8 gp70	-	51539	51279	261 | 86	hypothetical protein	-	-	-	-	-
vB_VpS_CA8 gp71	-	51768	51514	255 | 84	hypothetical protein	-	-	-	-	-
vB_VpS_CA8 gp72	-	52514	51768	747 | 248	hypothetical protein	-	-	-	-	-
vB_VpS_CA8 gp73	-	52685	52515	171 | 56	hypothetical protein	-	-	-	-	-
vB_VpS_CA8 gp74	-	53048	52755	294 | 97	hypothetical protein	-	-	-	-	-
vB_VpS_CA8 gp75	-	53465	53211	255 | 84	hypothetical protein	-	-	-	-	-
vB_VpS_CA8 gp76	-	53775	53449	327 | 108	hypothetical protein	-	-	-	-	-
vB_VpS_CA8 gp77	-	54146	53763	384 | 127	hypothetical protein	-	-	-	-	-
vB_VpS_CA8 gp78	-	54946	54650	297 | 98	hypothetical protein	-	-	-	-	-
vB_VpS_CA8	-	55334	55038	297 | 98	hypothetical protein	-	-	-	-	-

gp79										
vB_VpS_CA8 gp80	-	55920	55588	333 | 110	hypothetical protein	-	-	-	-	-
vB_VpS_CA8 gp81	-	56153	55932	222 | 73	hypothetical protein	-	-	-	-	-
vB_VpS_CA8 gp82	-	56727	56269	459 | 152	hypothetical protein	[*Pseudomonas* phage PaMx25]	YP_009603595.1	53.00%	1.00E-21	59.76%
vB_VpS_CA8 gp83	-	57074	56805	270 | 89	hypothetical protein	-	-	-	-	-
vB_VpS_CA8 gp84	-	58103	57618	486 | 161	hypothetical protein	-	-	-	-	-

Table. S4 ANIm percentage identity

	Prokaryotic dsDNA virus sp.	vB_VpaS_HCMJ	vB_VpaS_KF5	vB_VpaS_KF6	vB_VpaS_MAR10	Vibrio phage R01	Vibrio phage VpKK5	Vibrio phage VVP001	Vibrio vulnificus phage SSP002	vB_VpaS_KF3	vB_VpaS_KF4	vB_VpS_BA3	Vibrio phage VP06	vB_VpS_CA8
Prokaryotic dsDNA virus sp.	100.00%	0.00%	0.00%	0.00%	0.00%	0.00%	0.00%	0.00%	0.00%	0.00%	0.00%	0.00%	0.00%	0.00%
vB_VpaS_HCMJ	0.00%	100.00%	97.70%	98.20%	83.00%	82.96%	0.00%	97.75%	97.84%	85.31%	85.32%	0.00%	85.16%	0.00%
vB_VpaS_KF5	0.00%	97.70%	100.00%	97.52%	83.19%	83.40%	0.00%	98.07%	98.48%	85.43%	85.46%	0.00%	85.20%	0.00%
vB_VpaS_KF6	0.00%	98.20%	97.52%	100.00%	83.23%	83.04%	0.00%	97.53%	97.50%	86.32%	86.32%	0.00%	86.00%	0.00%
vB_VpaS_MAR10	0.00%	83.00%	83.19%	83.23%	100.00%	95.12%	0.00%	83.44%	83.05%	83.53%	83.51%	0.00%	83.20%	0.00%
Vibrio phage R01	0.00%	82.96%	83.40%	83.04%	95.12%	100.00%	0.00%	83.50%	83.19%	84.07%	83.82%	0.00%	83.59%	0.00%
Vibrio phage VpKK5	0.00%	0.00%	0.00%	0.00%	0.00%	0.00%	100.00%	0.00%	0.00%	0.00%	0.00%	0.00%	0.00%	0.00%
Vibrio phage VVP001	0.00%	97.75%	98.07%	97.53%	83.44%	83.50%	0.00%	100.00%	98.03%	85.29%	85.34%	0.00%	85.05%	0.00%
Vibrio vulnificus phage SSP002	0.00%	97.84%	98.48%	97.50%	83.05%	83.19%	0.00%	98.03%	100.00%	85.38%	85.38%	0.00%	85.15%	0.00%
vB_VpaS_KF3	0.00%	85.31%	85.43%	86.32%	83.53%	84.07%	0.00%	85.29%	85.38%	100.00%	99.99%	0.00%	96.43%	0.00%
vB_VpaS_KF4	0.00%	85.32%	85.46%	86.32%	83.51%	83.82%	0.00%	85.34%	85.38%	99.99%	100.00%	0.00%	96.42%	0.00%
vB_VpS_BA3	0.00%	0.00%	0.00%	0.00%	0.00%	0.00%	0.00%	0.00%	0.00%	0.00%	0.00%	100.00%	0.00%	96.48%
Vibrio phage VP06	0.00%	85.16%	85.20%	86.00%	83.20%	83.59%	0.00%	85.05%	85.15%	96.43%	96.42%	0.00%	100.00%	0.00%
vB_VpS_CA8	0.00%	0.00%	0.00%	0.00%	0.00%	0.00%	0.00%	0.00%	0.00%	0.00%	0.00%	96.48%	0.00%	100.00%

Table. S5 Characteristic of the vB_VpS_BA3 virion proteome identified by LC_MS/MS

Protein IDs	Product	Peptides	Sequence coverage [%]	Mol. weight [kDa]	Sequence length
vB_VpS_BA3 gp2	primase	4	9.6	89.653	793
vB_VpS_BA3 gp3	hypothetical protein	1	6.3	18.737	174
vB_VpS_BA3 gp4	hypothetical protein	3	25.1	21.036	191
vB_VpS_BA3 gp6	type II secretion system protein	3	21.3	14.965	136
vB_VpS_BA3 gp7	hypothetical protein	12	70.1	15.838	144
vB_VpS_BA3 gp8	ATPase	8	37.2	27.017	247
vB_VpS_BA3 gp9	multimodular transpeptidase-transglycosylase	2	9.4	33.942	307
vB_VpS_BA3 gp12	DNA/RNA helicase	2	5.4	63.701	570
vB_VpS_BA3 gp15	hypothetical protein	2	10.9	21.392	192
vB_VpS_BA3 gp17	transcriptional activator	3	15.1	23.232	218
vB_VpS_BA3 gp18	orgamic radical activating enzyme	1	12	25.927	234
vB_VpS_BA3 gp19	putative QueC-like protein	3	6.7	51.349	462
vB_VpS_BA3 gp23	type 1 GTP cyclohydrolase	5	32.9	22.763	207
vB_VpS_BA3 gp26	tRNA ribosyltransferase	3	25.3	36.32	316
vB_VpS_BA3 gp28	DNA polymerase beta subunit	4	29	36.12	324
vB_VpS_BA3 gp38	tail assembly protein	2	7.9	29.357	267
vB_VpS_BA3 gp39	tail assembly protein	4	10.6	61.619	545
vB_VpS_BA3 gp40	hypothetical protein	8	41.4	34.206	307
vB_VpS_BA3 gp41	hypothetical protein	5	31	30.211	274
vB_VpS_BA3 gp42	putative tape measure protein 1	5	13.1	88.411	800
vB_VpS_BA3 gp44	tail subunit	14	65.2	33.804	316
vB_VpS_BA3 gp45	hypothetical protein	1	12.2	15.34	139
vB_VpS_BA3 gp48	virion structural protein	2	30.9	18.362	175
vB_VpS_BA3 gp50	hypothetical protein	12	50.5	23.481	212
vB_VpS_BA3 gp51	Major capsid protein	29	88.6	38.009	352
vB_VpS_BA3 gp52	hypothetical protein	2	7.6	31.196	289
vB_VpS_BA3 gp54	structural phage protein	16	37.5	57.92	526
vB_VpS_BA3 gp55	terminase large subunit	2	12.1	60.359	522
vB_VpS_BA3 gp62	DNA ligase	5	16.4	40.839	353

**vB_VpS_BA3 gp66**	**hypothetical protein**	**2**	**13.1**	**17.384**	**153**
**vB_VpS_BA3 gp68**	**hypothetical protein**	**2**	**20.7**	**9.0853**	**82**
**vB_VpS_BA3 gp70**	**hypothetical protein**	**1**	**18.7**	**10.109**	**91**
**vB_VpS_BA3 gp74**	**hypothetical protein**	**2**	**16.6**	**17.881**	**151**
**vB_VpS_BA3 gp77**	**hypothetical protein**	**1**	**12.9**	**12.131**	**101**
**vB_VpS_BA3 gp81**	**hypothetical protein**	**2**	**23.6**	**12.614**	**110**
**vB_VpS_BA3 gp84**	**hypothetical protein**	**2**	**31.5**	**9.7161**	**89**
**vB_VpS_BA3 gp85**	**hypothetical protein**	**2**	**29.8**	**18.195**	**161**

Table. S6 Characteristic of the vB_VpS_CA8 virion proteome identified by LC_MS/MS

Protein IDs	Product	Peptides	Sequence coverage [%]	Mol. weight [kDa]	Sequence length
vB_VpS_CA8 gp2	primase	7	24	89.71	793
vB_VpS_CA8 gp7	hypothetical protein	6	46.5	15.837	144
vB_VpS_CA8 gp12	DNA/RNA helicase	5	13.2	63.75	569
vB_VpS_CA8 gp22	type 1 GTP cyclohydrolase	5	30.4	22.763	207
vB_VpS_CA8 gp26	hypothetical protein	1	21.4	7.9492	70
vB_VpS_CA8 gp34	tail assembly protein	31	53.6	92.875	842
vB_VpS_CA8 gp36	tail assembly protein	1	28.2	7.6528	71
vB_VpS_CA8 gp37	tail assembly protein	9	52.8	29.17	267
vB_VpS_CA8 gp38	tail assembly protein	20	53.5	61.074	548
vB_VpS_CA8 gp39	hypothetical protein	14	62.4	34.41	319
vB_VpS_CA8 gp40	hypothetical protein	13	63.2	30.929	277
vB_VpS_CA8 gp41	tail length tape-measure protein	45	52.8	88.318	799
vB_VpS_CA8 gp43	tail subunit	19	88.9	33.762	316
vB_VpS_CA8 gp44	hypothetical protein	2	14.4	15.382	139
vB_VpS_CA8 gp47	virion structural protein	7	50.9	18.376	175
vB_VpS_CA8 gp49	hypothetical protein	1	6.6	23.465	212
vB_VpS_CA8 gp50	major head protein	37	90.6	37.96	352
vB_VpS_CA8 gp52	minor head protein	11	40.6	44.57	387
vB_VpS_CA8 gp53	structural phage protein	26	64.6	57.906	526
vB_VpS_CA8 gp54	terminase large subunit	2	5.2	60.359	522
vB_VpS_CA8 gp65	hypothetical protein	3	18.3	17.384	153
vB_VpS_CA8 gp74	hypothetical protein	1	6.2	11.325	97
